# Insects in outer space: assessing the effects of microgravity on edible and model insect species for spaceflight food system

**DOI:** 10.3389/fphys.2025.1622401

**Published:** 2025-07-01

**Authors:** Roberto Guidetti, Annette Bruun Jensen, David Copplestone, Martina Heer, Paola Pittia, Lorena Rebecchi, Åsa Berggren

**Affiliations:** ^1^ Department of Life Sciences, University of Modena and Reggio Emilia, Modena, Italy; ^2^ Department of Plant and Environmental Sciences, University of Copenhagen, Frederiksberg, Denmark; ^3^ Biological and Environmental Sciences, University of Stirling, Stirling, United Kingdom; ^4^ IU International University of Applied Sciences, Bad Reichenhall, Germany; ^5^ Institute for Nutritional and Food Sciences, University of Bonn, Bonn, Germany; ^6^ Department of Bioscience and Technology for Food Agriculture and Environment, University of Teramo, Teramo, Italy; ^7^ Department of Ecology, Swedish University of Agricultural Sciences, Uppsala, Sweden

**Keywords:** edible, *Acheta domesticus*, *Tenebrio molitor*, *Apis mellifera*, space mission

## Abstract

Insects represent an extraordinary opportunity for human nutrition in extraterrestrial conditions. Therefore, the understanding of the effects of microgravity on the biology of edible insects in space conditions is essential for their use as food. Among the mostly used ones, the house cricket *Acheta domesticus*, the yellow mealworm *Tenebrio molitor*, and the honeybee *Apis mellifera* have been studied in microgravity conditions. Several other insects that are not used for food have been used as model species for space experiments. Considering that currently we are 75 years from the first space missions and a multitude of experiments, the results available on the effects of microgravity in insects are scarce and fragmented. Nevertheless, some data are available, the microgravity effects are species-specific, but generally the development and behaviour of individuals are not strongly affected. The developmental and metamorphic processes seem to be able to be completed in space and the reproduction and completion of life cycle for some species are possible. Negative effects from microgravity have been seen in the immune system and in physiology of some species. The results that we have so far from disparate studies, indicate that insect species may cope in space environments and thereby be part of making future long-term exploration missions possible.

## 1 Introduction

For long-term space-flight human explorations and for colonization of planets or satellites a constant and self-sustaining food supply is needed. Insects are already present in regular diets for billions of people and represent a sustainable food source (Omuse et al., 2024). The nutritional value of insects is extremely high. Indeed, they are an excellent source of complete protein, essential fatty acids, iron, zinc and B vitamins, all essential for growth and development of humans, with values often comparable to or higher than those of meat, fish and legumes (FAO, 2013; EFSA, 2015; Zhou et al., 2022). There are many evidences that proteins, lipids, and other elements of edible insects can replace traditional sources of nutrition (Orkusz, 2021; Zhou et al., 2022). Therefore, they may represent an extraordinary opportunity for human’s nutrition in extraterrestrial conditions (Dufour, 1981; Katayama et al., 2008; Jones, 2015; Li et al., 2016; Kok and Van Huis, 2021; Berggren et al., 2025). Thanks to their small size and lightweight, short life cycle, general ease of rearing, ability to withstand starvation and physical and chemical stresses, including reduced access to water, along with high food conversion rate and nutritional value associated to lower ethical concerns in respect to animal food sources, they are excellent candidates for such role. Moreover, insects are likely suitable as food source for humans living in isolated areas as they show high potential to be reared in bioregenerative life support systems (BLSS).

Among the various environmental factors that organisms in a BLSS will be exposed to in space conditions, the force of gravity is likely to be one of the most difficult to manage. Evolution of all living organisms on Earth has been under the constraints of the planet’s gravity force. Therefore, the understanding of the effects of microgravity in space on life cycles and physiology of reared insects is essential. As reported by Zhang et al. (2021) “the sheer inescapability of Earth’s gravitational pull has meant that its influence on Earth’s organisms is difficult to study.” For this reason, space flights and orbiting stations represent a fundamental laboratory to investigate how different forces of gravity (in this case its absence or reduction) can influence the life of organisms.

Many experiments have been performed in space to test the effects of microgravity in model invertebrate animals such as micro-invertebrates as nematodes (e.g., Honda et al., 2012; Kaplan et al., 2020), or tardigrades (Persson et al., 2011; Rebecchi et al., 2011), crustaceans (e.g., Gaubin et al., 1983) and macro-invertebrates such as molluscs (e.g., Balaban et al., 2011; Aseyev et al., 2017), echinoderms (e.g., Crawford and Jackson, 2002) and insects. Insects have been widely used in space experiments and much data are available on the effects of microgravity on these organisms, but the information remains scattered among the tested species, without a clear understanding on the effects for specific taxa.

## 2 Experiments with insects

Among the most common edible insects (Omuse et al., 2024) that were used in spaceflight experiments, the house cricket *Acheta domesticus* L. (Orthoptera, Gryllidae), the yellow mealworm *Tenebrio molitor* L. (Coleoptera, Tenebrionidae), and the honeybee *Apis mellifera* L. (Hymenoptera: Apidae) can be listed. The first two species are also approved by the European Food Safety Authority (EFSA) to be sold and eaten within Europe (European Commission, 2023).

### 2.1 House cricket (*Acheta domesticus*)

The experiments in space with this species have been mainly focused on the impact of microgravity (µG) on cellular processes. *Acheta domesticus* was used as model species within the project “Crickets in Space” (CRISP) and the potential gravity sensitive neuronal system was studied during several years (see Horn et al., 2007). Eggs, different larval stages and adults flew within spacecraft for different time periods, not exceeding 16 days (Förster et al., 1999; Horn et al., 2001; 2002; 2003; 2007; Kirschnik et al., 2002; Kirschnick and Horn, 2006). In-flight fertilization (after 7 days of space flight) occurred in orbit; after landing, flight eggs hatched 1.5 days earlier than the ground controls (Kirschnick and Horn, 2006; Horn et al., 2007). Morphological features of sensory cells and neurons of the newborn larvae were rarely seen to be affected by µG (Horn et al., 2007). Crickets of four developmental stages (eggs, I-, IV-, and, VI-larval stage), that flew in space for 16 days, showed a low susceptibility in their behavioural response to µG. However, the position-sensitive interneurons (related to movement coordination) activity revealed a significant sensitivity (Horn et al., 2003). Overall, microgravity seemed to rarely affect the morphological features of sensory cells and neurons development while the physiology of the position sensitive neuro system was significantly affected and their sensitivity reduced (Kirschnik et al., 2002; Horn et al., 2001; 2002; 2003; 2007). A change was seen, after 13 days of space flight, when the crickets appeared to be capable of normalising the development of their multi-channel gravity sensory system to their new gravitational environment (Förster et al., 1999). Microgravity is an environmental factor known to induce muscular atrophy in different species (Leonard and Albury, 2015). During simulated µG by clinorotation, a significant loss in muscle mass and enzymatic function was found in individuals of the species from day 1. Therefore, the natural histolysis of flight muscle that occurs in the house cricket development appears to happen sooner (Leonard and Albury, 2015). In general, the responses seen in *A. domesticus* to gravity reduction are likely to be adaptations related to physiological modifications and permanent changes (Horn et al., 2007). Short term µG conditions have not been found to impact development of a stable nervous function, as readaptation took place in individuals (Horn et al., 2007).

### 2.2 Mealworm (*Tenebrio molitor*) and other tenebrionids

Although *T. molitor* has been suggested as a food source for space travelers (Li et al., 2016; Jones, 2015), no experiments in orbit have involved this species. To elucidate the effects of low gravity on the larvae stage in the life cycle, mealworms were subject to a short duration experiment at approximately 0 G during parabolic flight. This treatment led to a 30% reduction of metamorphosed larvae (Davis, 1999), probably due to larvae dying.

Other tenebrionids have been used in space experiments, namely, the desert beetle (*Trigonoscelis gigas* Reitter, 1893), the red flour beetle (*Tribolium castaneum* Herbst, 1797), and the confused flour beetle (*Tribolium confusum* Du Val, 1863). The former one was used in several space flights and simulated gravity experiments to test if its activity rhythm and circadian functions were altered by µG (Alpatov, 1991; Alpatov et al., 1994; 1998; 2000; Hoban-Higgins et al., 1997; 2000; 2003). Both basic features of circadian rhythms and the expression of the clock responsible for these rhythms were found to be altered (Alpatov et al., 1994; Hoban-Higgins et al., 2003). In particular, the free-running period (i.e., animal is said to be free-running in an environment without time cues, the length of an animal’s day is determined by the period of its internal pacemaker) was significantly affected by both the µG and ambient light intensity, but with contrasting results: this period resulted in a longer time period according to Alpatov et al. (1998) and a shorter one in Hoban-Higgins et al. (2003).

The red flour beetle was used to study the occurrence of gravity-sensitive steps during oogenesis and embryogenesis. The results from two space flights of 6 and 10 days indicated that several aspects of the development and function of the reproductive system of females were not sensitive to microgravity (Bennett et al., 1994). In particular, μG did not affect the development of the female reproductive system, the viability of embryos, and inseminated females continued to lay fertile eggs, when back on land (i.e., there was no depletion of stored sperms) (Bennett et al., 1994).

For *T. confusum*, the experiments in µG (in orbit or simulated by fast-rotating clinorotation) suggested that embryogenesis was not gravity-sensitive, although some aspects of adult development could be (Cogoli, 1992; Bennett et al., 1994). The confused flour beetle successfully completed a full generation in space, although no precise quantitative data were obtained on mating competence and various aspects of development in this study (Miquel, 1984).

### 2.3 Honeybee (*Apis mellifera*)

Only three experiments have been performed with *A. mellifera* (Nelson and Peterson 1982; Vandenberg et al., 1985; Smith et al. (2021). During space flights, the bee flights were very brief without wingbeat (resulting in floating) and without control of attitude in any body axis. The control of their orientations for landing and movements prior to a rest period on a surface were very difficult (Nelson and Peterson, 1982). The post flight eggs hatchability showed contrasting results. According to Vandenberg et al. (1985), the eggs laid by the queen of a mature hive sent in space for 7 days failed to hatch after the mission’s return. By contrast, according to Smith et al. (2021), a queen was able to lay viable eggs after a shorter space flight and all observed eggs hatched into healthy offspring in the experiment.

### 2.4 Other insects

Other insects were used for space experiments. Another Apidae as the bumblebee (*Bombus ignites*) was studied in μG by parabolic flight, showing and altered flying behaviour (Yamashita et al., 2010). The lepidopteran velvet bean caterpillar moths (*Anticarsia gemmatalis*) and the dipteran common houseflies (*Musca domestica*) were studied by Nelson and Peterson (1982) in the same experiment with honeybees. In space conditions, moth flight activity was very uncontrolled and brief (less than 10 s), and the moths had some difficulty orienting themselves during landing. The uploaded moth pupae were able to successfully emergence to imago in space (Nelson and Peterson, 1982). Even the housefly flight activity was very brief (generally less than 1 s), but houseflies were capable of better controlling their pitch attitude, flight path, and landing process than moths and bees. From the preflight uploaded housefly pupae, the imago emerged successfully in space (Nelson and Peterson, 1982).

The hymenopteran pavement ants (*Tetramorium caespitum*) were studied to analyze how they performed collective search in µG (Countryman et al., 2015). Collective search is used by the ants to optimise the area covered by the group. In space conditions, the ants explored the surface less thoroughly with more convoluted routes, probably due to the difficulty in clinging to the surface. In fact, they often lost contact with the surface but showed a remarkable ability to regain it (Countryman et al., 2015). Between 2005–2010 the larvae of the chironomid *Polypedilum vanderplanki* Hinton, 1951 were utilized as a model organism in experiments on resistance of resting stages of invertebrates to space environment both inside and outside of ISS (Gusev et al., 2010a). The larvae in anhydrobiotic state survived after 18 months of direct exposure to outer space environments (Gusev et al., 2010b).

The stick insect *Carausius morosus* (Phasmatodea, Heteronemiidae) was used in different space missions as it was considered one of the classical models in developmental biology. Eggs at five different stages of development, representing different sensitivities to radiation and different capacities for regeneration, were tested. Eggs at five different stages flew for 7 days either at µG conditions or on the 1 G centrifuge to separate the effects of radiation and microgravity and to analyze the combined effects (Bücker et al., 1986). The early stages of development showed to be highly sensitive to radiations and to µG during development (e.g., µG reduced hatching rate). In some cases, the combined action of these factors amplified their negative effects and produce a high frequency of anormal larvae (Bücker et al., 1986). Eggs at different stages flew in other two missions for 9 and 13 days (Ushakov and Alpatov, 1992; Reitz et al., 1989; 1992). No significant changes in developmental time were detected although the hatching rate decreased, especially eggs with an age of 31 days (embryogenesis lasts 75–105 days) (Ushakov and Alpatov, 1992; Reitz et al., 1989; 1992). Six different ages of eggs further flew for 8 days (Reitz et al., 1995): a reduction of hatching rate was observed in agreement with previous studies, while morphological and developmental changes during embryo development were detected (in contrast with the previous missions), although some repair capacity seemed occur.

The insect most used in space experiments and the first animal survived in outer space (in a V-2 rocket in 1947; Beischer and Fregly, 1962) is the fruit fly *Drosophila melanogaster*. It represents a well-established spaceflight model organism: fruit flies have yielded significant information on the effects of microgravity in many different fields of physiology and behavioural and developmental sciences (for a review see Iyer et al., 2022). Overall, previous studies showed normal development of *Drosophila melanogaster* during flights, but evident structural and functional cardiac impairments, neurobehavioral deficits, suppression of immune system (with increase virulence for some pathogens; Gilbert et al., 2020), and alterations in gene expression profile were observed under spaceflight conditions (Iyer et al., 2022; Mhatre et al., 2022).

*Drosophila melanogaster* is the only insect for which it was tested the possibility to complete the life cycle and reproduction in space. Fruit flies were able to complete the life cycle (from zygote to mature adults) under space flight conditions, obtaining offsprings (Vernos et al., 1989; Ogneva et al., 2016; Marcu et al., 2011; Taylor et al., 2014). Size and number of eggs increased under microgravity conditions and life span decreased in males, but not in females (Marco et al., 1986; Vernos et al., 1989; Marthy, 2002). Still after 13 days of spaceflight, *D. melanogaster* maintained normal locomotor activity rhythm and sleep pattern (Ma et al., 2015), but circadian clock was affected. As the ability to keep a normal photoperiod may ease the effects of microgravity, a suitable photoperiod and lighting system (intensity, spectrum, and distribution) could offer powerful countermeasures for circadian and sleep disorders during spaces flights (Zhang et al., 2021).

## 3 Discussion

Despite the number of experiments carried out, the current knowledge of the effects of microgravity on insects remains very limited. This is due to several factors. In general, the science of life in space is constrained by the low number of space missions. For studies on insects in space this is further compounded by the fact that the studies of animals and their autecology (and not as human models) are very rare during any mission. To date, there is also no attempt among researchers to coordinate research and this has led to the use of several different model species and the analysis of different biological functions. These factors limit our knowledge and understanding of the effects of µG on insects and on their nutritional quality.

Several additional factors can also affect the results of experiments with insects in space conditions (Iyer et al., 2022): e.g., duration of the mission/experiment, facility and platforms used (e.g., parabolic flight, space flight, orbital station), payload/hardware and habitat designs, maintenance of environmental variables (e.g., temperature, humidity, gas composition), food supply (type and amount), and insect strains used in each study. The interpretation of the results can also be biased due to the different conditions in which the control animals are kept in. The animals that serve as Earth controls can also experience different conditions than their counterparts in space. For example, temperatures cannot always be well controlled or recorded during flights, or the launch/ascent accelerations (generating hypergravity) and the re-entry and landing conditions are factors that can affect the animals that go into space but not the ones that remain. Moreover, although not reported by many authors, the obtained data are frequently affected by pre- and post- flight difficulties in following the exact conditions to which the samples were subjected and kept. It is not always possible to control, check and record the pre-flight (i.e., time and environmental conditions spent by the samples between their delivery to the launchpad and the launch) and post flight (i.e., time and environmental conditions spent between landing and analyses) conditions of the samples. These are all factors that might lead to possible biases of the experimental results. When studying the effects of microgravity, there are technical constraints associated with space experiments that introduce complications with a possible compromission of results interpretation. It is not always clear whether changes detected in µG experiments reflect additional spaceflight-related stresses (e.g., temperature shifts, vibrational effects and radiation exposure) as opposed to the loss of gravitational force *per se* (Beckingham, 2010). In fact, the effects of space flight are due to the synergic effects of the environmental variables of the space environment in which microgravity and radiation play a central role. All above-mentioned factors and uncertainties make the results of different experiments, especially if they are few, difficult to compare.

Although the life cycles of insects are short, a limiting factor for studies is the duration of space missions (varying from a few minutes in parabolic flights to ≤50 days in orbital stations), which is usually shorter than the lifespan of an insect limiting full life-cycle studies.

Several are experiments on insects in space, but most of them are >25 years old, being carried out between 1960 and 2000, and for many of them information about environmental variables during the missions and the pre- and post-fights are not reported.

As the studies focused on several species, the consequence is that very little information on separate species is available. Additionally, very few aspects of the biology of the species were investigated. The results that we found from these studies are also sometimes contrasting and thus hard to draw general conclusions from. Therefore, there is not a clear overview of the effects of space microgravity on insects as a group.

Although data is scarce, we believe that some general conclusions can be made. The effects of microgravity are species-specific, but in general the development and behaviour of individuals are not strongly affected. The ability of insects to control their movement, especially flight, in microgravity varies greatly among species. Once started, the developmental (even inside the egg) and metamorphic processes seem to be able to be completed in space. Reproduction seems possible in space conditions, as is the completion of a life cycle (as shown for *D. melanogaster*), although negative effects can be seen in the immune system and for some physiological aspects. The habitat conditions in space can reduce some of the effects of microgravity, e.g., circadian and sleep disorders can be controlled by appropriate photoperiod and lighting systems. The current main knowledge gap of the effects of microgravity on insects are related to the absence of data on long term effects of microgravity and their effects on the complete insect life cycle. More than one generation of a species in microgravity condition has not been tested, and a general understanding of the effects of µG on the biology of species in microgravity is lacking.

Future investigations should focus on the possibility of target insect species to reproduce in space conditions (e.g., investigating the reproductive behaviour, gametes production, embryo development), to complete the life cycle (e.g., lifespan, growth), to produce several generations without reducing fitness (e.g., immune system response, infection risk, inbreeding problems), and to achieve the edible products with the composition in terms of macro- and micro-nutrients (e.g., proteins, lipids, vitamins and other essential elements) required for the use as food for astronauts and space travelers. Presently, the facilities to conduct long term experiments to grow insect species in isolated microgravity conditions are available (e.g., International Space Station). Facilities like these should be used to develop the much-needed knowledge for making sustainable BLSS a reality. The results that we have so far from disparate studies, indicate that insect species may cope in space environments and thereby be part of making future exploration missions possible.

## References

[B1] Alpatov A. M. (1991). Studies of circadian rhythms in space flight: some results and prospects. Physiologist 34 (1 Suppl. l), S145–S146. 2047416

[B2] Alpatov A. M. Chernyshov V. B. Zotov V. A. Reitveld W. J. (2000). Sand-desert tenebrionid beetle Trigonoscelis gigas reitter: a promising biological model for space chronobiology. Aerosp. Environ. Med. 34 (1), 58–61. 10732200

[B3] Alpatov A. M. Hoban-Higgins T. M. Fuller C. A. Lazarev A. O. Rietveld W. J. Tschernyshev V. B. (1998). Effects of microgravity on circadian rhythms in insects. J. gravitational physiology a J. Int. Soc. Gravitational Physiology 5 (1), P1–P4. 10.1016/0273-1177(92)90290-e 11542306

[B4] Alpatov A. M. Rietveld W. J. Oryntaeva L. B. (1994). Impact of microgravity and hypergravity on free‐running circadian rhythm of the desert beetle Trigonoscelis gigas reitt. Biol. rhythm Res. 25 (2), 168–177. 10.1080/09291019409360284 11541428

[B5] Aseyev N. Vinarskaya A. K. Roshchin M. Korshunova T. A. Malyshev A. Y. Zuzina A. B. (2017). Adaptive changes in the vestibular system of land snail to a 30-day spaceflight and readaptation on return to Earth. Front. Cell. Neurosci. 11, 348. 10.3389/fncel.2017.00348 29163058 PMC5672023

[B6] Balaban P. M. Malyshev A. Y. Ierusalimsky V. N. Aseyev N. Korshunova T. A. Bravarenko N. I. (2011). Functional changes in the snail statocyst system elicited by microgravity. PLoS One 6 (3), e17710. 10.1371/journal.pone.0017710 21479267 PMC3066201

[B7] Beckingham K. M. (2010). Synergy between stresses: an interaction between spaceflight‐associated conditions and the microgravity response. Mol. Ecol. 19, 4105–4107. 10.1111/j.1365-294x.2010.04799.x 25241407

[B8] Beischer D. E. Fregly A. R. (1962). Animals and man in space. A chronology and annotated bibliography through the year 1960 US Nav. Sch. Aviat. Med. ONR TR ACR-64 (AD0272581).

[B9] Bennett R. L. Abbott M. K. Denell R. E. (1994). Insect gravitational biology: ground‐based and shuttle flight experiments using the beetle *Tribolium castaneum* . J. Exp. Zoology 269 (3), 242–252. 10.1002/jez.1402690309 11536636

[B10] Berggren Å. Jensen A. B. Copplestone D. Guidetti R. Heer M. Pittia P. (2025). Insects as food for space travel and planetary colonisations. Front. Physiol. 10.3389/fphys.2025.1622401 PMC12259422 40666115

[B11] Bücker H. Facius R. Horneck G. Reitz G. Graul E. H. Berger H. (1986). Embryogenesis and organogenesis of *Carausius morosus* under spaceflight conditions. Adv. Space Res. 6 (12), 115–124. 10.1016/0273-1177(86)90074-8 11537809

[B12] Cogoli M. (1992). The fast rotating clinostat: a history of its use in gravitational biology and a comparison of ground-based and flight experiment results. ASGSB Bullettin 5, 59–67. 11537642

[B13] Countryman S. M. Stumpe M. C. Crow S. P. Adler F. R. Greene M. J. Vonshak M. (2015). Collective search by ants in microgravity. Front. Ecol. Evol. 3, 25. 10.3389/fevo.2015.00025

[B65] Crawford B. J. Jackson D. (2002). Effect of microgravity on the swimming behaviour of larvae of the starfish Pisaster ochraceus. Canad. J. Zool. 80 (12), 2218–2225. 10.1139/z02-206

[B14] Davis A. M. (1999). “The effects of variable gravity on the life cycle of *Tenebrio molitor* ,” in The founding convention of the mars society (Boulder, CO), 767–771.

[B15] Dufour P. A. (1981). Insects: a nutritional alternative (No. NASA 1.26: 169056).

[B66] Du Val C. J. (1863). Genera des coléoptères d'Europe: comprenant leur classification en familles naturelles, la description de tous les genres, des tableaux dichotomiques destinés à faciliter l'étude, le catalogue de toutes les espèces, de nombreux dessins au trait de caractères. A. Deyrolle Naturaliste 3, 181 du Catalogue.

[B16] EFSA (2015). Scientific opinion on an alternative method for the hygienic treatment of bovine colostrum through a series of filtration steps. EFSA J. 13 (10), 4139. 10.2903/j.efsa.2015.4139 32313571 PMC7163586

[B17] European Commission (2023). Available online at: https://food.ec.europa.eu/safety/novel-food/authorisations/approval-insect-novel-food_en.

[B18] FAO (2013). Edible insects: future prospects for food and feed security. Available online at: https://www.fao.org/3/i3253e/i3253e.pdf.

[B19] Förster S. Sebastian C. Horn E. R. (1999). “Functional regeneration of the cercal gravity sensory system of crickets (*Acheta domesticus*) in the absence of gravity,”. Göttingen neurobiology report 1999. Editor Elsner N. (Stuttgart, New York: Thieme), II, 800.

[B20] Gaubin Y. Planel H. Gasset G. Pianezzi B. Delpoux M. Clegg J. (1983). Results on Artemia cysts, lettuce and tobacco seeds in the Biobloc 4 experiment flown aboard the Soviet Biosatellite Cosmos 1129. Adv. Space Res. 3 (8), 135–140. 10.1016/0273-1177(83)90183-7 11542741

[B21] Gilbert R. Torres M. Clemens R. Hateley S. Hosamani R. Wade W. (2020). Spaceflight and simulated microgravity conditions increase virulence of *Serratia marcescens* in the *Drosophila melanogaster* infection model. npj Microgravity 6 (1), 4. 10.1038/s41526-019-0091-2 32047838 PMC7000411

[B22] Gusev O. Nakahara Y. Sychev V. Levinskikh M. Novikova N. Alexeev V. (2010a). An anhydrobiotic insect, *Polypedilum vanderplanki* as a tool for astrobiology. Space Utiliz. Res 24, 306–309.

[B23] Gusev O. Sakashita T. Sychev V. Novikova N. Sugimoto M. Malyutina L. (2010b). The sleeping chironomid: an insect survived 18 months of exposure to outer space. 38th COSPAR Sci. Assem. 38, 9.

[B67] Herbst J. F. W. (1797). Natursystem aller bekannten in undauslandischen insekten, als eine Fortsetzung der vom Buffonschen Naturgeschichte. Berlin: Der kafer pts, 4–7.

[B24] Hoban-Higgins T. M. (2000). Effects of gravity on insect circadian rhythmicity. NTRS - NASA Tech. Rep. Serv. Available online at: https://ntrs.nasa.gov/citations/20010000284.

[B25] Hoban-Higgins T. M. Alpatov A. M. Wassmer G. T. Reitveld W. J. Fuller C. A. (1997). Response of insect activity rhythms to altered gravitational environments. J. Gravit. Physiol. 4 (2), P109–P110. 11540665

[B26] Hoban-Higgins T. M. Alpatov A. M. Wassmer G. T. Rietveld W. J. Fuller C. A. (2003). Gravity and light effects on the circadian clock of a desert beetle, *Trigonoscelis gigas* . J. insect physiology 49 (7), 671–675. 10.1016/s0022-1910(03)00068-4 12837319

[B27] Honda Y. Higashibata A. Matsunaga Y. Yonezawa Y. Kawano T. Higashitani A. (2012). Genes down-regulated in spaceflight are involved in the control of longevity in *Caenorhabditis elegans* . Sci. Rep. 2 (1), 487. 10.1038/srep00487 22768380 PMC3390002

[B28] Horn E. Agricola H. Böser S. Förster S. Kämper G. Riewe P. (2002). Crickets in space: morphological, physiological and behavioral alterations induced by space flight and hypergravity. Adv. Space Res. 30 (4), 819–828. 10.1016/s0273-1177(01)00642-1 12530388

[B29] Horn E. Böser S. Förster S. Riewe P. Sebastian C. Agricola H. (2001). Crickets in space. Acta Astronaut. 49 (3-10), 345–363. 10.1016/s0094-5765(01)00111-4 11669122

[B30] Horn E. R. Dournon C. Frippiat J. P. Marco R. Böser S. Kirschnick U. (2007). Development of neuronal and sensorimotor systems in the absence of gravity: neurobiological research on four soyuz taxi flights to the international space station. Microgravity Sci. Technol. 19, 164–169. 10.1007/bf02919474

[B31] Horn E. R. Kämper G. Neubert J. Buckery J. C. Homick J. L. (2003). “The development of an insect gravity sensory system in space (crikets in space),” in The neurola spacelab mission neuroscience research in space (Huston, Texas: Johnson Space Center).

[B32] Iyer J. Mhatre S. D. Gilbert R. Bhattacharya S. (2022). Multi-system responses to altered gravity and spaceflight: insights from *Drosophila melanogaster* . Neurosci. Biobehav. Rev. 142, 104880. 10.1016/j.neubiorev.2022.104880 36126744

[B33] Jones R. S. (2015). Space diet: daily mealworm (*Tenebrio molitor*) harvest on a multigenerational spaceship. J. Interdiscip. Sci. Top. 4.

[B34] Kaplan F. Shapiro-Ilan D. Schiller K. C. (2020). Dynamics of entomopathogenic nematode foraging and infectivity in microgravity. npj Microgravity 6 (1), 20. 10.1038/s41526-020-00110-y 32818149 PMC7418002

[B35] Katayama N. Ishikawa Y. Takaoki M. Yamashita M. Nakayama S. Kiguchi K. (2008). Entomophagy: a key to space agriculture. Adv. Space Res. 41 (5), 701–705. 10.1016/j.asr.2007.01.027

[B36] Kirschnick U. Horn E. R. (2006). Effects of altered gravity on identified peptidergic neurons of the cricket *Acheta domesticus* . Gravitational Space Biol. Bull. 19 (2), 135–136.

[B37] Kirschnik U. Horn E. Agricola H.-J. (2002). The influence of microgravity on the morphology of identified cerebral neurons in a cricket (*Acheta domesticus*). Life Space Life Earth 501, 137–138. 14703671

[B38] Kok R. Van Huis A. (2021). Insect food in space. J. Insects as Food Feed 7 (1), 1–4. 10.3920/jiff2021.x001

[B39] Leonard K. C. Albury A. N. J. (2015). To fly or not to fly? An observation of the effects of simulated microgravity on the flight muscles of the house cricket, *Acheta domesticus* . Explorations, 22–27.

[B40] Li L. Stasiak M. Li L. Xie B. Fu Y. Gidzinski D. (2016). Rearing *Tenebrio molitor* in BLSS: dietary fiber affects larval growth, development, and respiration characteristics. Acta Astronaut. 118, 130–136. 10.1016/j.actaastro.2015.10.003

[B41] Ma L. Ma J. Xu K. (2015). Effect of spaceflight on the circadian rhythm, lifespan and gene expression of *Drosophila melanogaster* . PLoS One 10 (3), e0121600. 10.1371/journal.pone.0121600 25798821 PMC4370389

[B43] Marco R. Vernós I. Gonzalez J. Calleja M. (1986). Embryogenesis and aging of *Drosophila melanogaster* flown in the space shuttle. Naturwissenschaften 73, 431–432. 10.1007/bf00367288 3093897

[B44] Marcu O. Lera M. P. Sanchez M. E. Levic E. Higgins L. A. Shmygelska A. (2011). Innate immune responses of *Drosophila melanogaster* are altered by spaceflight. PloS one 6 (1), e15361. 10.1371/journal.pone.0015361 21264297 PMC3019151

[B45] Marthy H. J. (2002). Developmental biology of animal models under varied gravity conditions: a review. Vie et Milieu/Life and Environment, 52 (4), 149–166.

[B46] Mhatre S. D. Iyer J. Petereit J. Dolling-Boreham R. M. Tyryshkina A. Paul A. M. (2022). Artificial gravity partially protects space-induced neurological deficits in *Drosophila melanogaster* . Cell Rep. 40 (10), 111279. 10.1016/j.celrep.2022.111279 36070701 PMC10503492

[B47] Miquel J. (1984). “Effects of microgravity and hypergravity on invertebrate development,” in NASA developmental biology workshop. NASA technical memorandum 86756. Editors Souza K. Halstead T. (Ames Research Center), 7–34.

[B48] Nelson T. E. Peterson J. R. (1982). NSTA-NASA shuttle student involvement project. Experiment results: insect flight observation at zero gravity (No. NASA-CR-173028).

[B49] Ogneva I. V. Belyakin S. N. Sarantseva S. V. (2016). The development of *Drosophila melanogaster* under different duration space flight and subsequent adaptation to earth gravity. PLoS One 11 (11), e0166885. 10.1371/journal.pone.0166885 27861601 PMC5115863

[B50] Omuse E. R. Tonnang H. E. Yusuf A. A. Machekano H. Egonyu J. P. Kimathi E. (2024). The global atlas of edible insects: analysis of diversity and commonality contributing to food systems and sustainability. Sci. Rep. 14 (1), 5045. 10.1038/s41598-024-55603-7 38424443 PMC10904393

[B51] Orkusz A. (2021). Edible insects versus meat—nutritional comparison: knowledge of their composition is the key to good health. Nutrients 13 (4), 1207. 10.3390/nu13041207 33917531 PMC8067469

[B52] Persson D. Halberg K. A. Jørgensen A. Ricci C. Møbjerg N. Kristensen R. M. (2011). Extreme stress tolerance in tardigrades: surviving space conditions in low earth orbit. J. Zoological Syst. Evol. Res. 49, 90–97. 10.1111/j.1439-0469.2010.00605.x

[B53] Rebecchi L. Altiero T. Cesari M. Bertolani R. Rizzo A. M. Corsetto P. A. (2011). Resistance of the anhydrobiotic eutardigrade *Paramacrobiotus richtersi* to space flight (LIFE–TARSE mission on FOTON‐M3). J. Zoological Syst. Evol. Res. 49, 98–103. 10.1111/j.1439-0469.2010.00606.x

[B68] Reitter E. (1893). Achter beitrag zur coleopteren-fauna des russischen reiche. Arrhaphipterus schelkownikoffi n. sp. Wien. Entomol. Zeit. 12, 109–114.

[B54] Reitz G. Beaujean R. Hiendl O. C. Rüther W. Pross H. D. Kost M. (1995). Radiation and microgravity effects observed in the IML-1 MOROSUS experiment (10-D MOROSUS). Biorack Spacelab IML-1 1162, 187.

[B55] Reitz G. Bücker H. Facius R. Horneck G. Graul E. H. Berger H. (1989). Influence of cosmic radiation and/or microgravity on development of *Carausius morosus* . Adv. Space Res. 9 (10), 161–173. 10.1016/0273-1177(89)90435-3 11537289

[B56] Reitz G. Bücker H. Lindberg C. Hiendl O. C. Rüther W. Graul E. H. (1992). Radiation and microgravity effects observed in the insect system *Carausius morosus* . Int. J. Radiat. Appl. 20 (1), 233–239. 10.1016/1359-0189(92)90103-3

[B57] Smith R. S. H. Kraemer F. Bader C. Smith M. Weber A. Simone-Finstrom M. (2021). A rapid fabrication methodology for payload modules, piloted for the observation of queen honey bees (*Apis mellifera*) in microgravity. Gravitational Space Res. 9 (1), 104–114. 10.2478/gsr-2021-0008

[B58] Taylor K. Kleinhesselink K. George M. D. Morgan R. Smallwood T. Hammonds A. S. (2014). Toll mediated infection response is altered by gravity and spaceflight in *Drosophila* . PLoS One 9 (1), e86485. 10.1371/journal.pone.0086485 24475130 PMC3901686

[B59] Ushakov I. A. Alpatov A. M. (1992). Possible mechanism of microgravity impact on *Carausius morosus* ontogenesis. Adv. Space Res. 12 (1), 153–155. 10.1016/0273-1177(92)90278-6 11536952

[B60] Vandenberg J. D. Massie D. R. Shimanuki H. Peterson J. R. Poskevich D. M. (1985). Survival, behavior and comb construction by honey bees, *Apis Mellifera*, in zero gravity aboard NASA Shuttle Mission STS-13. Apidologie 16, 369–384. 10.1051/apido:19850402

[B61] Vernos I. Gonzalez-Jurado J. Calleja M. Marco R. (1989). Microgravity effects on the oogenesis and development of embryos of *Drosophila melanogaster* laid in the Spaceshuttle during the Biorack experiment (ESA). Int. J. Dev. Biol. 33 (2), 213–226. 2518159

[B62] Yamashita M. Hashimoto H. Mitsuhata M. Sasaki M. (2010). Flight performance of bumble bee as a possible pollinator in space agriculture under partial gravity. 38th COSPAR Sci. Assem. 38, 6.

[B63] Zhang H. Wang Y. Zhang Z. Zhang L. Tang C. Sun B. (2021). Alterations in the activity and sleep of *Drosophila melanogaster* under simulated microgravity. npj Microgravity 7 (1), 27. 10.1038/s41526-021-00157-5 34294729 PMC8298474

[B64] Zhou Y. Wang D. Zhou S. Duan H. Guo J. Yan W. (2022). Nutritional composition, health benefits, and application value of edible insects: a review. Foods 11 (24), 3961. 10.3390/foods11243961 36553703 PMC9777846

